# Is It Still Double Edged? Not for University Students’ Development of Moral Reasoning and Video Game Play

**DOI:** 10.3389/fpsyg.2020.01313

**Published:** 2020-06-11

**Authors:** Sarah E. Hodge, Jacqui Taylor, John McAlaney

**Affiliations:** Department of Psychology, Bournemouth University, Poole, United Kingdom

**Keywords:** university sample, moral development, moral reasoning, Kohlberg, video games, computer games, cross-sectional

## Abstract

Previous research with video game play and moral development with adolescents, found both positive and negative relationships. This study aimed to extend this research to explore moral development and video game play with university students. One hundred and thirty-five undergraduate students (*M* = 20.29, SD = 2.70) took part in an online survey. The results suggested higher moral reasoning for participants who described themselves as gamers and those which do not play, compared those who play but do not identify as gamers. It was suggested that males had higher moral scores and more mature reasoning than females. The results of a regression analysis suggested that there were no significant predictors for moral development from either game play or the demographic variables. The findings suggest that moral development could be less influenced by sex, age, and video game play factors such as video game content and amount of game play, than was previously thought for this age group.

## Introduction

Gaming is a popular pastime among adults; with an average of 4.8 hours spent a week playing online and those aged 18–34 (known as millennial gamers) making up about 40% of gamers, with the industry worth reported (from transactions in 2019) to be $43.4 billion (ESA, 2019). Furthermore, the gaming industry is growing and is expected to be worth $90 billion in 2020 (WePC, 2020). There have been long standing concerns over the effects of media on the users, with violent video games receiving much attention from research and media (American Psychological Association [APA], 2015; Bowman, 2016). Much of the previous research with video games has focused mainly on adult samples and post-game outcomes; with the American Psychological Association reported that, overall only some short-term effects of violent games but acknowledge there are methodological issues within the research (American Psychological Association [APA], 2015). Further criticisms of the research methods and outcomes have been documented (Brown v. Entertainment Merchants Association [EMA], 2011; Elson and Ferguson, 2014). A recent statement from the APA concludes how the results for video games and aggression are still mixed, and that more validation is needed for these claims (Ferguson et al., 2017). This was further reflected in recent recommendations for the research process and methodology for violent video games and aggression (Hilgard et al., 2017; McCarthy and Elson, 2018; Elson, 2019; Elson et al., 2019). The research around violent video games causing aggression remains contentious, especially from the reported publication bias and the validity of meta-analyses (Ferguson, 2007, 2015; Furuya-Kanamori and Doi, 2016; Elson, 2019).

Longitudinal research has been carried out to explore the long term role of aggression with violent video games. In one study with children, a risk factor for pro-social behavior was found with those who played competitive games for 8 h or more a week, but there was no association found with violent video games (Lobel et al., 2017). Furthermore, longitudinal research carried out with adolescents in Singapore found no effects of violent video games (Ferguson and Wang, 2019), similar findings to that of previous research with adolescents (Ferguson et al., 2012). A study with adults aged 18–45 were either assigned to play GTA, the Sims or no video game, for 2 months. The results suggested no significant effects on aggression or mental health (Kühn et al., 2018). Likewise, no relationship was found between playing violent games being aggressive and future consumption of violent video games for the young adults aged 18–21, but a relationship between future consumption and violent video games was found for the 14–17 year olds (Breuer et al., 2015). This study highlights the importance of exploring the long-term relationship/effects of video game play with adults as well as the potential differences between young adults and adolescents.

Since then research has started to explore other psychological factors that could be related to video game play such as, morality (Eden et al., 2012). Joeckel et al. (2012) measured upholding or violating the moral foundations through in-game scenarios with the Moral Foundation Questionnaire (MFQ; Graham et al., 2008). The results from two age groups, adolescents (aged range 12–15) and older adults (age range 49–86) suggested that the moral foundations were more likely to be upheld, with the adolescent group being the most random in their in-game decisions, demonstrating age differences between the groups. Joeckel et al. (2013) carried out a follow-up study with adolescents (age range 12–14) and found similar results to the previous study where foundations were more likely to be upheld. However, it would have been interesting to include older young people to explore if there were any further age differences between adolescents and young adults.

Previous research on video games and morality with university samples have found that participants (age range 18–29) demonstrated moral sensitivity, through feelings of guilt when playing as terrorists (Grizzard et al., 2014). Furthermore, Weaver and Lewis (2012) found that in-game moral decisions of participants (age range 18–24) were suggested to reflect real life moral decisions. Contrary to this it has been found that morality (participants aged between 17 and 25) can be disengaged in video game play (Hartmann and Vorderer, 2010). All of these three studies have used an adult university sample demonstrating the potential role of video games and morality for this age group. However, a potential important factor which has had less focus could be the developmental factors, for example, how age of the participant and their moral development relates to in-game moral decisions and video game play. Echoed in a review which highlighted the need for more research on moral development of young people and video game play (Jin et al., 2017); as many previous studies have explored other factors of morality such as in-game decisions (e.g., Weaver and Lewis, 2012). Thus, there is much scope for research to explore moral development, video game play with university students.

Kohlberg’s (1971) theory of moral development, which was developed further by Gibbs et al. (1992) into the Sociomoral Reflection Measure (SRM), suggests that university students’ moral reasoning is still developing. Hence, as moral reasoning is still developing and together with the popularity of game play for this age group questions remain around; if moral development has a relationship with university students game play. Previous research by Bajovic (2013) used the SRM with adolescents (aged 13–14) and only found a small negative relationship with length of time playing violent video games and SRM scores. Hodge et al. (2019) found positive and negative relationships with moral development also using the SRM measure with an adolescent sample (aged between 11 and 18). The results of Hodge et al. (2019) also suggested that number of genres played had a significant positive relationship with moral reasoning scores. Gaming is a popular pastime for adults and previous research with adolescents found relationship between video game play and moral reasoning, questions remain regarding the relationship between video game play and moral reasoning for young adults. Therefore, there was much scope to extend this research and follow-up with an older age group with young adults, particularly university students. The previous research has highlighted a gap with how individuals interact with media across the life span to include the role of development. Much of this research with moral development has focused on younger age groups, but there is scope to explore moral development with university students, one such example was a study which found a traditional ethics board game could be supporting moral development (Huang and Ho, 2018). Demonstrating the importance of extending the research of moral development with university samples to video game play. The study aims to build on previous research with the SRM measure through exploring if there is a relationship between moral development and self-reported video game play among young adult samples (university students).

## Materials and Methods

### Participants

Full ethical approval was obtained through the Universities ethics committee. Undergraduate University participants were recruited through opportunity sampling and with (49%) recruited from the SONA system for psychology students to gain course credits. All participants gave written informed consent in accordance with the Declaration of Helsinki. A total of 135 participants were sampled.

### Procedure

The study was an online study as a follow-up to Hodge et al. (2019), participants completed the survey through the online survey platform SurveyMonkey. The order participants completed the measures were: first asked the demographics questions (free school meals, ethnicity, age, and sex), then they completed the moral reasoning measure, followed by the video game play questions. To reduce some demand characteristics minor deception was used in the study with all questions worded in a neutral way to reduce any stereotype threat. Specifically, participants were asked about their gameplay from their experiences rather than in a competitive/competence context (Steele and Aronson, 1995; Steele, 1997; Casad and Bryant, 2016). Participants were told the study was about decision-making in video games rather than morality. In the debrief participants were informed of the studies focus and asked to tick a box to confirm that they were still happy to be included in the study.

### Measures

#### Sociomoral Reflection Measure–Short Form (SRM-SF)

The SRM was used to measure moral development as the range of this measure includes adults (see Table 1). The measure consists of four stages of moral development, stages 1–2 were classed as immature reasoning and stages 3–4 were classed as mature reasoning (Gibbs et al., 1992). The norms for university student’s moral development is stage 3 reasoning, the beginning of mature moral reasoning, see Table 1 (Gibbs et al., 1992). This means that university students have the potential to develop morality into transition stage 3 (3.5) and stage 4 (final stage). Plus, the results of this study could be compared with previous research using the measure (Bajovic, 2013; Hodge et al., 2019). SRM reports a good concurrent validity of *r* = 0.69 and test retest reliability of *r* = 0.88 (Gibbs et al., 1992).

**TABLE 1:** SRM norms of Moral A adapted from Gibbs et al. (1992).

School Age United Kingdom (American)	Age	Global stage	Score boundary of global stage	Maturity
Year 5 (fourth grade)	10.05	2	1.75–2.25	Immature
Year 7 (sixth grade)	12.06	2 (3)	2.26–2.49	Immature
Year 9 (eighth grade)	14.11	3 (2)	2.50–2.74	Immature
Sixth form (high school)	17.30	3	2.75–3.25	Mature
University	19.18	3	2.75–3.25	Mature
Adult	50.66	4 (3)	3.50–3.74	Mature

*Adapted from “N, Mean SRM-SF, Mean global stage, age, and SES by sample” by Gibbs et al. (1992, p. 40).*

#### Video Game Play

The questions on self-reported video game play were the same questions that were used in previous research as they were in-depth and could be compared (Hodge et al., 2019). These questions included many aspects of game play including gaming status (if participants played video games), length of time playing, number of genres played (from a list), average content rating, and identifying as a gamer, Average content rating and if the game contained a moral narrative was calculated from the mean ESRB (2015) and PEGI (2015) rating of favorite games (see Appendix A). An additional question was added for this sample, to ask if participants identified as gamers, to explore how participants describe their game play. See Tables 3, 4 for the list variables and descriptive statistics (for further information on the video game play questions see Hodge, 2018; Hodge et al., 2019).

**TABLE 2:** The SRM development of the sample.

Global stage	Score boundary of global stage	Maturity	Frequency (*n* = 120)	Percent
3 (2) lower 3	2.50–2.74	Immature	5	4.2
3	2.75–3.25	Mature	60	50.0
3 (4) upper 3	3.26–2.49	Mature	38	31.7
4 (3) lower 4	3.50–3.74	Mature	16	13.3
4	3.75–4.00	Mature	1	0.8

**TABLE 3:** Descriptive statistics for sex and continuous video game play variables.

Gaming variables continuous	*N*	*M*	SD	*t/U*	*Df/z*	*r*
Years playing Range = 0–17						
Male	41	13.17	3.23			
Female	17	9.24	4.96			
*Total*	58	12.02	4.18	3.02	21.87	0.42**
Number of genres played Range = 0–19						
Male	48	10.52	4.98			
Female	72	4.49	4.11			
*Total*	120	6.90	5.36	7.23	118	0.55***
Content rating Range = 0–5						
Male	45	2.91	0.74			
Female	58	1.79	1.11			
*Total*	103	2.28	1.11	6.13	98.85	0.51***
Engagement Range = 0–38						
Male	48	16.96	7.64			
Female	72	9.83	9.08			
*Total*	120	12.68	9.19	4.48	118	0.39***
Length of time Range = 0–37.5						
Male	48	13.57	9.15			
Female	72	4.22	5.20			
*Total*	120	7.96	8.39	547.50	−6.35	−0.58***

r is the effect size reported. Length of time (Mdn = 6) was not normally distributed (kurtosis) a Mann–Whitney U was carried out for this variable with independent t-tests carried out on the rest of the gaming variables. **p < 0.01, ***p < 0.001.

**TABLE 4:** Descriptive statistics for sex and categorical video game play variables.

Gaming variables categorical	Yes	No	Total	χ^2^ (1)	Odds ratio
Gaming status					
Male	47	1	48		
Female	64	8	72		
*Total*	111	9	120	3.38	–
Gamers					
Male	43	5	48		
Female	17	55	72		
*Total*	60	60	120	50.14	27.82***
Non-gamer					
Male	4	44	48		
Female	47	25	72		
*Total*	51	69	120	38.22	0.05***
Violent					
Male	38	7	45		
Female	18	31	49		
*Total*	56	38	94	22.17	9.35***
Mature					
Male	36	9	45		
Female	16	33	49		
*Total*	52	42	94	10.80	8.25**
Moral Narrative					
Male	38	15	53		
Female	7	34	41		
*Total*	45	49	94	27.64	12.30***
GTA					
Male	19	26	45		
Female	6	43	43		
*Total*	25	69	94	10.80	5.24**
COD					
Male	6	39	45		
Female	3	46	49		
*Total*	9	85	94	1.41	–

Odds ratio is the effect size reported and is not calculated for non-significant results. **p < 0.01, ***p < 0.001.

#### Game Engagement Questionnaire

The Game Engagement Questionnaire (GEQ) was included to measure the level of engagement and how participants usually feel when playing video games. The measures consisted of 19 questions and scored; Yes = 2 Maybe = 1 and No = 0. The maximum score on the measure is 38 α = 0.85 (Brockmyer et al., 2009; Fox and Brockmyer, 2013).

### Data

The data for the SRM were analyzed through matching and identifying participants’ response to Criterion Justification (CJ) within a stage of moral development and moral type for each of the 11 questions. The eleven questions consisted of five themes: contract and truth, affiliation, life, property and law, and legal justice. All scorable responses would have yielded a type A moral score which was an average stage of moral development. Those that did yield a score could have also demonstrated Moral type B which was identified if participants referred to two of three components (balancing, fundamental valuing, and conscience) in their responses. Gibbs et al. (1992) suggest the Moral type A show an embedding of moral concepts whereas Moral type B is an expression of moral concepts. Not all responses yielded a scorable response; for this sample 14 responses did not yield a scorable response (for further information on scoring the SRM responses please see, Gibbs et al., 1992).

## Results

This study aims to explore the relationship between moral development (SRM scores) and video game play for university students (Hodge et al., 2015). Of the 135 participants (*M* = 20.29, SD = 2.70), just under half were male (42%) and the majority of the sample had a White (Scottish, Irish English or other) background (85%). Free school meals were taken as a measure of social economic status (SES) with 25% receiving free school meals at some point during schooling. One participant aged 41 years was subsequently removed as an outlier on the basis of their age being much higher than the next oldest participant and they would be classed as a different generation of gamer GEN X (ESA, 2019). The sample updated age range 17–27 years (*M* = 20.04, SD = 1.83). There were no significant differences for SES [*t*(38.51) = −0.18, *p* > 0.05] and ethnicity [*t*(118) = 0.91, *p* > 0.05] with SRM scores and were not included in further analysis.

### Moral Development

Table 2 shows that only five participants moral reasoning was classed as immature. A high majority of the sample were suggested to have mature moral reasoning, with only 14% of the sample scoring into stage four with less than 1% at the highest stage of development.

### Video Game Play

Tables 3, 4 reports the game play by the participants by sex, with Table 3 suggesting significant differences between males and females game play; with males playing video games for more years, more types of genres, higher rated content and for a longer amount of time. All continuous variables reported a medium to large effect size, with length of time reporting the highest effect size.

Table 4 suggests that there were significant sex differences between most of the game play variables; with males 27 times more likely to identify as a gamer, were less than 1 time more likely to identify as a non-gamer, between 8 and 9 times more likely to play games with violent and mature content, 12 times more likely to play games with a moral narrative and 5 times more likely to play Grand Theft Auto (GTA) (Rockstar, 1997–2019). Gaming status and Call of Duty (COD) (Activision, 2005–2019) did not have significant sex differences.

Table 5 suggests that males had a higher global stage of moral development compared to females. There were also differences in the stage of development by game play; those that did not play video games and identified as gamers had a higher global stage of moral development. From the gaming status and gamer variables it was suggested that a third group emerged which was those that did play (yes to gaming status) but did not identify as gamers, a dummy variable was created to represent this group and labeled as non-gamers. Table 5 suggests that the non-gamers had a lower stage of moral reasoning (stage 3) than the gamers and non-players. A one-way ANOVA was conducted on the three group (non-player, non-gamers, gamers) with SRM scores *F*(2,117) 3.05, *p* = 0.051, but these differences were not significant and *post hoc* tests were not carried out.

**TABLE 5:** SRM scores, sex, gaming status, gamers, and non-gamers.

	*N*	*M*	SD	Global stage
Sex				
Males	48	3.27	0.24	3 (4)
Females	72	3.21	0.23	3
Gaming status				
Yes	111	3.23	0.24	3
No	9	3.29	0.20	3 (4)
Gamer				
Yes	60	3.28	0.25	3 (4)
No	60	3.19	0.21	3
Dummy variable				
Non-gamers	51	3.17	0.21	3

*The parentheses for global stage indicates if the scores are in the upper or lower score boundary of the stage, see Table 2.*

### Moral Development and Video Game Play

A multiple linear regression was carried out on moral development scores and video game play. As recommended by Field (2009) to support generalizability of the results and suitability of the analysis the regression assumptions were checked before carrying out the analysis.

Table 6 show that none of the variables predicted moral reasoning and development from the SRM scores, this include the demographic (age and sex) and moral type (A and B) as well as the gaming variables (see Appendix B).

**TABLE 6:** Predictors of SRM scores.

Variable^a^	B	95% CI [LL,UL]	SE B	β	VIF
Constant	3.02	(1.93, 4.07)	0.53		
Moral type	0.12	(−0.02, 0.26)	0.07	0.25	1.23
Sex	−0.04	(−0.23, 0.16)	0.10	−0.08	2.26
Age	0.01	(−0.03, 0.05)	0.02	0.09	1.22
Gamers	−0.04	(−0.25, 0.17)	0.10	−0.08	2.76
Years playing	−0.01	(−0.02, −0.02)	0.01	−0.08	1.61
Length of time	0.00	(−0.02. 0.01)	0.01	−0.16	2.76
Number of genres played	0.01	(−0.02, 0.03)	0.01	0.12	3.14
Content rating	−0.01	(−0.12, 0.11)	0.06	−0.05	3.96
Engagement (GEQ)	0.00	(−0.01, 0.01)	0.01	−0.08	2.04
GTA	0.16	(−0.03, 0.35)	0.10	0.31	1.82
Moral narrative	−0.20	(−0.40, 0.03)	0.11	−0.42	2.83
*R*^2^	0.23				
Δ*R*^2^	0.03				

*Forced entry method was used as no hierarchy was applied to the input of the gaming variables. ^a^Gaming status was removed by SPSS from the model due to low numbers. Data labels: moral type 1 = A; 2 = B. Sex 1 = male; 2 = female, Gaming status, gamer, moral narrative 1 = Yes; 2 = No. Due to multicollinearity; non-gamers, violent, and mature predictors were removed from the model. COD was also removed from the model due to low numbers of participants in the yes group. CI, confidence interval at 95% for B. VIF are within the suggested acceptable range (Thompson et al., 2017).*

## Discussion

This study explored the relationship between moral development (SRM scores) and video game play for university students. The results suggested no predictors of moral development for either video game play or demographic variables (age and sex). This suggests that moral development and reasoning may not be related to video game play, as often presumed. Although moral development was not predicted by video games there were some differences and trends between participants. Males had higher moral scores than females, and there were significant differences between males and females game play with most of the gaming variables; except playing COD and gaming status (playing the video games). The video game play variables suggested that there were different sub-groups of video game play style emerging; those that did not play (non-players), those that did play but did not identify as gamers (non-gamers) and those that identified as gamers. When the sub-groups are compared, gamers and non-players had higher moral score than the non-gamers.

### Implications

The results of moral development and sex differences between males and females in university age students was similar to Hodge et al. (2019) based on adolescents; with males on average having higher moral scores than females by a global stage. The results could suggest that males may prefer/find it easier to express morality through reasoning rather than emotions; due to stereotyping threat and specifically around male emotional suppression (Steele and Aronson, 1995; Steele, 1997; Cai et al., 2016). This sex difference could have been reflected in the result of previous research where moral sensitivity was found after playing a video game as the majority of the sample was female and moral sensitivity was measured through moral emotions, including guilt (Grizzard et al., 2014). Therefore, this could suggest that sex differences are due to different measures of morality (emotional or reasoning) being used, and moral reasoning may be more neutral in its approach to measuring morality when considering the role of stereotypes (Steele and Aronson, 1995; Steele, 1997; Cai et al., 2016). Furthermore, some previous research suggested that violent video games were related to young people having higher scores of psychopathy traits which was suggested to impact on interpersonal-affective skills (Kimmig et al., 2018). However, this could be related to stereotype threat regarding participants showing interpersonal-affective skills rather than psychopathy (Steele and Aronson, 1995; Steele, 1997). Particularly as other research has found no such link between psychopathy and shooter games (Smith et al., 2018). Overall, the combination of the previous research highlights the importance of how morality is measured; in that including both moral reasoning and sensitivity measures in research could support measuring morality more representatively.

Overall the results of moral development suggested a gradual progression for the university sample. This gradual development could be due to the small variability in the age range of the sample; university samples tend to recruit from 2 to 3 year groups (first, second, and placement years). Interestingly, recent debates have suggested that adolescence should be extended into the early 20s (Sawyer et al., 2018). Sawyer et al. (2018) suggest that major developmental transitions occur later and a new age range of 10–24 would better encompass these changes. If this new age range of adolescence was used in this research, it could support the proposal that morality is still developing. On the other hand, previous research found age differences as 14–17 year olds violent video game play did predict future consumption of violent video games, but this was not the case for the adult group aged 18–21, Breuer et al. (2015). Plus, research with participants aged 11–18 suggested this age group could be very different from university students; in that moral development was suggested to have a relationship with video game play and a transition took place between the ages of 12–14 (Hodge et al., 2019). Furthermore, Gibbs et al. (1992) suggests the role of physical maturation for young adolescent was related to females reaching higher scores before males. Although, most of the physical maturation has taken place for university students, cognitive and emotional development is still on-going (Sawyer et al., 2018) which parallels with moral development specifically, moral reasoning. It could be that moral development has peaked, which was suggested by Kohlberg’s theory and not all individuals will reach the higher stages; this was addressed when Gibbs et al. (1992) developed the SRM measure as the latter stages (5–6) did not transfer well into stages of development and that only 4 clear stages emerged. An alternative explanation is that moral development continues between university and adults as suggested by SRM, see Table 1 (Gibbs et al., 1992).

Therefore, moral development could be supported and encouraged as stage four moral reasoning relates to wider systems and standards of society (Gibbs et al., 1992). This could be represented in university and many jobs that require functioning in a larger social system to challenge moral reasoning to develop from immediate peer groups to wider social groups. Interestingly, some video games could also simulate these stage four concepts, as they can require players to function in complex social environments and systems such as guilds and in massively multiplayer online (MMO) games (Khoo, 2012). It could be suggested that stage four reasoning relates to utilitarian theory, which encourages moral reasoning in the context of considering the majority and the wider social group (Bentham, 1789). One way to measure this is through the trolley problem e.g., would one life be sacrificed to save a group of people (Thomson, 1985). The trolley problem has been recreated through a virtual environment where it was found that most participants (aged between 18 and 29 *M* = 19.61) selected the utilitarian option (to save more lives), as opposed to deontological (not sacrificing a life) (Navarrete et al., 2012). The design of video games has been suggested to have the potential to be morally engaging and support higher moral reasoning and maturity (Schrier, 2019; Staines et al., 2019). It could be suggested that due to the complexity of stage four reasoning that only specific games/design/game play (e.g., games with a moral narrative and role playing games with large social groups) would relate to stage 4, suggesting the importance of gathering many game play variables to understand participants game play habits. Specifically, there could be certain aspects and specific variables of video game play which relates to and supports morality, such as games that include guilds, but more research is needed to explore this potential relationship. Future technological developments with video games could allow for further sophistication and complexity which could support engaging higher levels of moral reasoning; whereas currently they may only be able to support certain stages of moral development. It is also acknowledged that there are other factors (e.g., environmental factors) which contribute to moral development (Gibbs et al., 1992).

This lack of relationship between moral development and video game play is an interesting finding when compared to previous research which has suggested a moral presence for in-game morality (e.g., Weaver and Lewis, 2012) as it could suggest a separation between the game and real-life morality. This relates to the concept of moral management in game play where it has been suggested that there is a separation of actions between the game and real-life, especially for violent behaviors (Klimmt et al., 2006). Moral disengagement is also an important mechanism which could underly how morality, specifically moral reasoning may relate to video games. Moral disengagement may especially relate to moral reasoning as it is a form of cognitive distortion (Bandura et al., 1996). Previous research with violent video games suggests moral disengagement in video game play supports enjoyment of the game (Hartmann and Vorderer, 2010) and other research suggest that violent video games were related to disinhibition and psychopathy traits (Kimmig et al., 2018).Therefore, the potential implications for morality and moral reasoning is that moral disengagement could become more proficient with age and/or increasing usage of this mechanism. Furthermore, previous research exploring moral disengagement factors in video games found that moral justification, which relates to moral reasoning, had one of the highest frequencies in video games (Hartmann et al., 2014). This could suggest a high prevalence of moral reasoning during video game play but happens through the process of moral disengagement which could explain the null findings. There are also implications for the model of intuitive morality and exemplars (MIME) model (Tamborini, 2011, 2012) suggests both short term and long term processes with media, and this theory could be further expanded to include developmental factors; such as does age relate to how sophisticated the deliberative processes are, especially for complex/mature content as well as the role of age and selection of media and video game play.

Similar to previous research with video game play (i.e., Hodge et al., 2019), the results of this study has implications for sex and gameplay as it was suggested that males were reporting more game play than females on the majority of game play variables, with the biggest difference being the length of time playing and identifying as a gamer. This demonstrates the importance of gathering video game play variables in research to understand previous game play habits of the participants. Length of time has been reported previously in research and would seem to be an important variable to include, especially given the sex difference reported by males. Also, these differences between the game play style could show the potential sub types and diversity within video game players, both how they identify themselves and what they play which has been previously identified (Galyonkin, 2015; Kaye, 2019). Sex differences in video game play have been found in previous research (Ferguson et al., 2015; Hartmann et al., 2015; Hodge et al., 2019) and the observation that they are still found in this age group could be due to similar stereotypes and potential stigma for female video game players (Kaye and Pennington, 2016). Also, previous research has suggested that stereotype threat can be represented by participants reporting the opposite behavior to the stereotype to disconfirm it (Pennington et al., 2016), which did not seem to be evident in this current research, but we acknowledge that stereotype threat could still be influencing participants responses. Especially, as previous research found that violent video games could be facilitating ideas and beliefs around traditional masculinity from both male and female participants (Blackburn and Scharrer, 2019). Although it should be noted that a high majority of the participants both male and female were playing video games which was similar to the Entertainment Software Association statistics (ESA, 2019) and demonstrates that gaming is an activity which both males and females engage with.

Although the research reported null findings, these findings suggest some important implications for age, development, and the interaction with media. In line with previous longitudinal research, which found no relationship between aggression and video game play for adults (Kühn et al., 2018). The implication of the content suggests the importance and effectiveness of the rating board systems, such as PEGI (2015) and ESRB (2015). University students were suggested not to have relationships between video game play and moral development, compared to previous research with adolescents which did suggest a relationship with moral development (Bajovic, 2013; Hodge et al., 2019). Therefore, demonstrating the importance and value of these rating boards for younger audiences; as it was the younger audience which lead to concerns and controversy for video games and lead to the introduction of these systems (McKernan, 2013). This lack of relationship between moral development and video game play for adults; supports the appropriateness of the age ratings to be for those aged under 18 as those over 18 could be less susceptible to influences from the content and guidance is not needed.

### Limitations

An alternative explanation for the higher moral scores in this research could be related to those who play more video games, in this case suggested by the data to be males and gamers, engaging more positively with the research and thus creating a bias. Cross-sectional designs have limitations, participants are compared to other participants rather than their own development over time in longitudinal studies. Smith et al. (2018) particularly highlight this need for more longitudinal research. Only one rater was used to code the SRM data and although experienced with the measure, more raters could have supported inter-rater reliability. Around half of the sample were psychology students and their game play maybe different from other courses and students who have different game play styles. It should also be acknowledged that potentially university students may have different moral reasoning compared to samples of the same age that did not attend university. It could be argued that either university students have the opportunity to challenge their thinking and reasoning whereas, those who did not attend university may get the opportunity to develop moral reasoning from life experiences and/or complex work experiences (Gibbs et al., 2007). Likewise, the way norms are developed has been found to become similar in institutions such as schools (Brugman et al., 2003), which could also happen at universities and could be addressed through involving more than one institution in the data collection. The majority of the sample were also white and therefore ethnic diversity within the sample was low and most did not receive free school meals suggesting low diversity for SES. It is also acknowledged that there are many other factors from an individual’s environment that will directly and indirectly relate to moral development (Gibbs et al., 1992). Another limitation of the research is that this study focused on the long-term influences of moral development, specifically moral reasoning and video game play but it is acknowledged that there could be potential shorter term influences of video games and other aspects of morality that could have been measured such as moral emotions and intuitive morality (Haidt and Joseph, 2004). Furthermore, self-report measures are always constrained by their nature of being self-report and how accurately participants answer. Due to the nature of this study self-report was required and actions were taken to reduce some biases (see section “Procedure”).

## Future Research and Conclusion

Exploring morality within a virtual context gives the opportunity to explore moral processes in a different context but can also develop further understanding of real-life moral development. Future research could consider the role of moral development in video game play, as moral development could act as both a mediating (i.e., what aspects of game play relate to moral development) and moderating variable (i.e., the role of age and strength of the relationship). There is also scope for future research to explore further how moral development could be supported for adults, particularly how university students could have moral learning and development supported through their studies at university. There could potentially be a development shift where young people will develop into higher stage 3/stage 4, which future research could explore with a larger age range of adult samples. There are also implications for how morality should be measured virtually, including if measures are appropriate (i.e., measuring moral emotion or reasoning) and sensitivity (i.e., to allow for moral developmental differences) and the differences between exploring long-term and short-term effects/relationships. In conclusion, we suggest that moral development does not have a significant relationship with any of video game play and demographic variables for the university sample.

## Data Availability Statement

The raw data supporting the conclusions of this article will be made available by the authors, without undue reservation, to any qualified researcher.

## Ethics Statement

The studies involving human participants were reviewed and approved by the Bournemouth University – Science, Technology & Health Research Ethics Committee. The patients/participants provided their written informed consent to participate in this study.

## Author Contributions

SH contributed to the conception, design, data collection, and analysis. All authors contributed to the manuscript revision, read, and approved the submitted version.

## Conflict of Interest

The authors declare that the research was conducted in the absence of any commercial or financial relationships that could be construed as a potential conflict of interest.

## Appendix

### Appendix A

**TABLE A1:** Rating Scale of video game content from ESRB and PEGI.

Scale	ESRB	PEGI
0	Early childhood	N/A
1	Everyone	3
2	Everyone + 10	7
3	Teen	12
4	Mature	16–18
5	Adult only	N/A

**TABLE A2:** The descriptive statistics and list of genres played by participants.

Genre (with some examples given in brackets for participants)	*N*	%
Action (Lego series and LittleBigPlanet)	47	39
Action-adventure (Grand Theft Auto series and Assassin’s Creed)	61	51
Adventure (Tomb Raider, Prince of Persia, and Uncharted)	46	38
Simulation, vehicle and racing (The Sims, Need for Speed, and Spore)	74	62
Strategy/puzzle (Monkey Island, Tetris, Portal, and Minecraft)	67	56
Role playing games (Fable, Skyrim, and Diablo)	48	40
Shooter (Battlefield, Spec Ops: The line, Halo, and Call of Duty)	47	39
Horror/survival (Resident Evil, Silent Hill, and Evil Within)	25	21
Violent games (Bioshock, Hitman, and Gears of War)	44	37
Platform games (Mario, Sonic the Hedgehog, and Smash Brothers)	63	53
Fighting/beat them up (Mortal Kombat, Street Fighter, Dead or Alive, and WWE)	33	28
Mini games and applications (apps) (Candy Crush and Clash of Clans)	72	60
Online/social games (World of Warcraft and Farmville)	35	29
Sport games and activity (FIFA and Kinect Sports, and Kinect Adventures)	30	25
Non-mainstream games (This War of Mine)	31	26
Games based on films (X Men, Alien, and Shadow of Mordor)	30	25
Dance, music, and fitness games (Dance Central, Guitar Hero, and Zumba)	42	35
Arcade games (House of the Dead and TimeCrisis)	30	25
Other	3	3
None	9	8

*Participants could select as many of the genres as applicable. Simulation, vehicle and racing, and mini games and applications (apps) were played the most by over 60% of participants followed by strategy/puzzle, action-adventure, and platform games which were played by half of the participants (51–56%). The genre played the least by participants was horror/survival (21%).*

### Appendix B

**TABLE B1:** Correlations matrix of SRM scores, demographics and game play variables.

	1	2	3	4	5	6	7	8	9	10	11	12
1. SRM	–											
2. Moral type	0.29***	–										
3. Sex	–0.14	0.10	–									
4. Age	0.12	0.03	−0.25***	–								
5. Gaming status	0.07	0.04	0.17***	−0.02**	–							
6. Gamers	−0.19*	–0.03	0.65**	−0.20***	0.29***	–						
7. Years playing	0.06	–0.14	−0.43***	0.32***		−0.38**	–					
8. Length of time	0.09	–0.01	−0.55***	0.00***	−0.27**	−0.67***	0.27*	–				
9. Number of genres played	0.12	–0.10	−0.55***	0.09***	−0.36***	−0.68***	0.49***	0.71***	–			
10. Content rating	0.14	0.04	−0.50***	0.09***	−0.51***	−0.60***	0.34**	0.56***	0.62***	–		
11. Engagement (GEQ)	0.07	0.07	−0.39***	0.10***	−0.34***	−0.51***	0.20	0.47***	0.53***	0.62***	–	
12. GTA	0.14	0.30**	0.34***	−0.11**		0.18**	–0.14	–0.08	−0.25**	−0.44***	–0.16	–
13. Moral narrative	−0.22*	0.09	0.54***	−0.10***		0.50***	−0.33**	−0.44**	−0.53***	−0.74***	−0.38***	0.53***

**p < 0.05, **p < 0.01, ***p < 0.001.*

## References

[B1] Activision (2005–2019). *Call of Duty (COD)*. Santa Monica, CA: Activision.

[B2] American Psychological Association [APA] (2015). *Task Force on Violent Media Technical Report on the Review of the Violent Video Game Literature.* Available online at: http://www.apa.org/news/press/releases/2015/08/technical-violent-games.pdf (accessed January 12, 2016).

[B3] Bajovic M. (2013). Violent video gaming and moral reasoning in adolescents: is there an association? *Educ. Media Int.* 50 177–191. 10.1080/09523987.2013.836367

[B4] Bandura A. Barbaranelli C. Caprara G. V. Pastorelli C. (1996). Mechanisms of moral disengagement in the exercise of moral agency. *J. Personal. Soc. Psychol.* 71 364–374. 10.1037/0022-3514.71.2.364

[B5] Bentham J. (1789). *1996. The Collected Works of Jeremy Bentham: An Introduction to the Principles of Morals and Legislation.* Oxford: Clarendon Press.

[B6] Blackburn G. Scharrer E. (2019). Video game playing and beliefs about masculinity among male and female emerging adults. *Sex Roles* 80 310–324. 10.1007/s11199-018-0934-4

[B7] Bowman N. D. (2016). “The rise (and refinement) of moral panic,” in *The Video Game Debate: Unravelling the Physical, Social, and Psychological Effects of Video Games*, ed. Kowert R. T., (New York, NY: Routledge), 22–38. 10.4324/9781315736495-2

[B8] Breuer J. Vogelgesang J. Quandt T. Festl R. (2015). Violent video games and physical aggression: evidence for a selection effect among adolescents. *Psychol. Pop. Media Cult.* 4 305–328. 10.1037/ppm0000035

[B9] Brockmyer J. H. Fox C. M. Curtiss K. A. McBroom E. Burkhart K. M. Pidruzny J. N. (2009). The development of the Game Engagement Questionnaire: a measure of engagement in video game-playing. *J. Exp. Soc. Psychol.* 45 624–634. 10.1016/j.jesp.2009.02.016

[B10] Brown v. Entertainment Merchants Association [EMA] (2011). *Supreme Court of the United States.* Available online at: https://www.supremecourt.gov/opinions/10pdf/08-1448.pdf (accessed January 17, 2020).

[B11] Brugman D. Heymans P. G. Boom J. Podolskij A. I. Karabanova O. Idobaeva O. (2003). Perception of moral atmosphere in school and norm transgressive behaviour in adolescents: an intervention study. *Int. J. Behav. Dev.* 27 289–300. 10.1080/01650250244000272

[B12] Cai A. Lou Y. Long Q. Yuan J. (2016). The sex differences in regulating unpleasant emotion by expressive suppression: extraversion matters. *Front. Psychol.* 7:1011. 10.3389/fpsyg.2016.01011 27458408 PMC4935688

[B13] Casad B. J. Bryant W. J. (2016). Addressing stereotype threat is critical to diversity and inclusion in organizational psychology. *Front. Psychol.* 7:8. 10.3389/fpsyg.2016.00008 26834681 PMC4718987

[B14] Eden A. Grizzard M. Lewis R. J. (2012). “Moral psychology and media theory: historical and emerging viewpoints,” in *Media and the Moral Mind*, ed. Tamborini R., (London: Routledge), 1–25.

[B15] Elson M. (2019). Examining psychological science through systematic meta-method analysis: a call for research. *Adv. Methods Pract. Psychol. Sci.* 2 350–363. 10.31234/osf.io/cfbzt

[B16] Elson M. Ferguson C. J. (2014). Twenty-five years of research on violence in digital games and aggression: empirical evidence, perspectives, and a debate gone astray. *Eur. Psychol.* 19 33–46. 10.1027/1016-9040/a000147

[B17] Elson M. Huff M. Utz S. (2019). Meta science on peer review: testing the effects of study originality and statistical significance in a field experiment. *MetaArXiv* [Preprints]. 10.31222/osf.io/dkuhs

[B18] ESA (2019). *Essential Facts About the Computer and Video Game Industry Sales, Demographics, and Usage Data.* Available online at: https://www.theesa.com/wp-content/uploads/2019/05/ESA_Essential_facts_2019_final.pdf (accessed 2019).

[B19] ESRB (2015). *Entrainment Software Rating Board.* Available online at: http://www.esrb.org/ratings/ (accessed June 2015).

[B20] Ferguson C. Klisinan D. Hogg J. L. Wilson J. Markey P. Przybylski A. (2017). *News Media, Public Education and Public Policy Committee.* Available online at: https://div46amplifier.com/2017/06/12/news-media-public-education-and-public-policy-committee/ (accessed January 20, 2020).

[B21] Ferguson C. J. (2007). Evidence for publication bias in video game violence effects literature: a meta-analytic review. *Aggress. Violent Behav.* 12 470–482. 10.1016/j.avb.2007.01.001

[B22] Ferguson C. J. (2015). Do Angry Birds make for angry children? A meta-analysis of video game influences on children’s and adolescents’ aggression, mental health, prosocial behavior, and academic performance. *Perspect. Psychol. Sci.* 10, 646–666. 10.1177/1745691615592234 26386002

[B23] Ferguson C. J. Wang J. C. (2019). Aggressive video games are not a risk factor for future aggression in youth: a longitudinal study. *J. Youth Adolesc.* 48 1439–1451. 10.1007/s10964-019-01069-0 31273603

[B24] Ferguson C. J. San Miguel C. Garza A. Jerabeck J. M. (2012). A longitudinal test of video game violence influences on dating and aggression: a 3-year longitudinal study of adolescents. *J. Psychiatr. Res.* 46 141–146. 10.1016/j.jpsychires.2011.10.014 22099867

[B25] Ferguson C. J. Trigani B. Pilato S. Miller S. Foley K. Barr H. (2015). Violent video games don’t increase hostility in teens, but they do stress girls out. *Psych. Q.* 87 49–56. 10.1007/s11126-015-9361-7 25896582

[B26] Field A. (2009). *Discovering Statistics Using SPSS.* Thousand Oaks, CA: Sage publications.

[B27] Fox C. M. Brockmyer J. H. (2013). The development of the game engagement questionnaire: a measure of engagement in video game playing: response to reviews. *Interact. Comput.*

[B28] Furuya-Kanamori L. Doi S. A. (2016). Angry birds, angry children, and angry meta-analysts: a reanalysis. *Perspect. Psychol. Sci.* 11 408–414. 10.1177/1745691616635599 27217253

[B29] Galyonkin S. (2015). *Your Target Audience Doesn’t Exist: Why You Shouldn’t Talk About “MOBA Audience”, “Core Gamers”, “Female Gamers” and instead Think Smaller.* Available online at: https://medium.com/steam-spy/your-target-audience-doesn-t-exist-999b78aa77ae#.vutuyivik (accessed July 9, 2019).

[B30] Gibbs J. C. Basinger K. S. Fuller D. (1992). *Moral Maturity: Measuring the Development of Sociomoral Reflection.* Hillsdale, NJ: Erlbaum.

[B31] Gibbs J. C. Basinger K. S. Grime R. L. Snarey J. R. (2007). Moral judgment development across cultures: revisiting Kohlberg’s universality claims. *Dev. Rev.* 27 443–500. 10.1016/j.dr.2007.04.001

[B32] Graham J. Haidt J. Nosek B. (2008). *The Moral Foundations Questionnaire (MFQ).* Available online at: http://www.moralfoundations.org/questionnaires (accessed July 1, 2015).

[B33] Grizzard M. Tamborini R. Lewis R. J. Wang L. Prabhu S. (2014). Being bad in a video game can make us more morally sensitive. *CyberPsychol. Behav. Soc. Network.* 17 499–504. 10.1089/cyber.2013.0658 24950172

[B34] Haidt J. Joseph C. (2004). Intuitive ethics: how innately prepared intuitions generate culturally variable virtues. *Daedalus* 133 55–66. 10.1162/0011526042365555 32495221

[B35] Hartmann T. Krakowiak K. M. Tsay-Vogel M. (2014). How violent video games communicate violence: a literature review and content analysis of moral disengagement factors. *Commun. Monogr.* 81 310–332. 10.1080/03637751.2014.922206

[B36] Hartmann T. Möller I. Krause C. (2015). Factors underlying male and female use of violent video games. *New Media Soc.* 17 1777–1794. 10.1177/1461444814533067

[B37] Hartmann T. Vorderer P. (2010). It’s okay to shoot a character: moral disengagement in violent video games. *J. Commun.* 60 94–119. 10.1111/j.1460-2466.2009.01459.x

[B38] Hilgard J. Engelhardt C. R. Rouder J. N. (2017). Overstated evidence for short-term effects of violent games on affect and behavior: a reanalysis of Anderson et al. (2010). *Psychol. Bull.* 143 757–774. 10.1037/bul0000074 28639810

[B39] Hodge S. E. (2018). *Press trigger for Morality: An Exploration Into the Role of Moral Development, Moral Decision-Making AND Video Game Play.* Doctor of PhilosophycThesis, Bournemouth University, Bournemouth.

[B40] Hodge S. E. Taylor J. McAlaney J. (2015). *Moral Development Scores Video Game Play University Students.* 1.

[B41] Hodge S. E. Taylor J. McAlaney J. (2019). The relationships between moral development, video game play and age of adolescents. *Front. Psychol. Hum. Media Interact.* 10:28. 10.3389/fpsyg.2019.00028 PMC6357883 30740072

[B42] Huang W. Ho J. C. (2018). Improving moral reasoning among college students: a game-based learning approach. *Interact. Learn. Environ.* 26 583–596. 10.1080/10494820.2017.1374979

[B43] Jin Y. Ma M. Hua D. Coward S. (2017). “Games for mental and moral development of youth: a review of empirical studies,” in *Joint International Conference on Serious Games*, Cham: Springer, 245–258. 10.1007/978-3-319-70111-0_23

[B44] Joeckel S. Bowman N. D. Dogruel L. (2012). Gut or game? The influence of moral intuitions on decisions in video games. *Media Psychol.* 15 460–485. 10.1080/15213269.2012.727218

[B45] Joeckel S. Bowman N. D. Dogruel L. (2013). The influence of adolescents’ moral salience on actions and entertainment experience in interactive media. *J. Child. Media* 7 480–506. 10.1080/17482798.2013.781513

[B46] Kaye L. K. (2019). “Gaming classifications and player demographics,” in *Oxford Handbook of Cyberpsychology*, eds Attrill-Smith A. Fullwood C. Kuss D. Keep M., (Oxford: Oxford University Press).

[B47] Kaye L. K. Pennington C. R. (2016). “Girls can’t play”: the effects of stereotype threat on females’ gaming performance. *Comput. Hum. Behav.* 59 202–209. 10.1016/j.chb.2016.02.020

[B48] Khoo A. (2012). Video games as moral educators? *Asia Pacific J. Educ.* 32 416–429. 10.1080/02188791.2012.738638

[B49] Kimmig A. C. S. Andringa G. Derntl B. (2018). Potential adverse effects of violent video gaming: interpersonal-affective traits are rather impaired than disinhibition in young adults. *Front. Psychol.* 9:736. 10.3389/fpsyg.2018.00736 29867689 PMC5964217

[B50] Klimmt C. Schmid H. Nosper A. Hartmann T. Vorderer P. (2006). How players manage moral concerns to make video game violence enjoyable. *Communications* 31 309–328.

[B51] Kohlberg L. (1971). “Stages of moral development as a basis for moral education,” in *Moral education; Interdisciplinary Approaches*, eds Beck C. M. Crittenden B. S. Sullivan E. V., (Toronto: University of Toronto Press), 23–92.

[B52] Kühn S. Kugler D. T. Schmalen K. Weichenberger M. Witt C. Gallinat J. (2018). Does playing violent video games cause aggression? A longitudinal intervention study. *Mol. Psychiatry* 24 1220–1234. 10.1038/s41380-018-0031-7 29535447 PMC6756088

[B53] Lobel A. Engels R. C. Stone L. L. Burk W. J. Granic I. (2017). Video gaming and children’s psychosocial wellbeing: a longitudinal study. *J. Youth Adolesc.* 46 884–897. 10.1007/s10964-017-0646-z 28224404 PMC5346125

[B54] McCarthy R. J. Elson M. (2018). A conceptual review of lab-based aggression paradigms. *Collabra Psychol.* 4 1–12. 10.1525/collabra.10430637365

[B55] McKernan B. (2013). The morality of play video game coverage in the New York Times From 1980 to 2010. *Games Cult.* 8 307–329. 10.1177/1555412013493133

[B56] Navarrete C. D. McDonald M. M. Mott M. L. Asher B. (2012). Virtual morality: emotion and action in a simulated three-dimensional “trolley problem”. *Emotion* 12 364–370. 10.1037/a0025561 22103331

[B57] PEGI (2015). *Pan European Game Information.* Available online at: https://pegi.info/page/pegi-age-ratings (accessed June 2015).

[B58] Pennington C. R. Heim D. Levy A. R. Larkin D. T. (2016). Twenty years of stereotype threat research: a review of psychological mediators. *PLoS One* 11:e0146487. 10.1371/journal.pone.0146487 26752551 PMC4713435

[B59] Rockstar, (1997- 2019). *Grand Theft Auto (GTA).* New York, NY: Rockstar.

[B60] Sawyer S. M. Azzopardi P. S. Wickremarathne D. Patton G. C. (2018). The age of adolescence. *Lancet Child Adolesc. Health* 2 223–228. 30169257 10.1016/S2352-4642(18)30022-1

[B61] Schrier K. (2019). Designing games for moral learning and knowledge building. *Games Cult.* 14 306–343. 10.1177/1555412017711514

[B62] Smith S. Ferguson C. Beaver K. (2018). A longitudinal analysis of shooter games and their relationship with conduct disorder and cself-reported delinquency. *Int. J. Law Psychiatry* 58 48–53. 10.1016/j.ijlp.2018.02.008 29853012

[B63] Staines D. Formosa P. Ryan M. (2019). Morality play: a model for developing games of moral expertise. *Games Cult.* 14 410–429. 10.1177/1555412017729596

[B64] Steele C. M. (1997). A threat in the air: how stereotypes shape intellectual identity and performance. *Am. Psychol.* 52 613–629. 10.1037/0003-066x.52.6.613 9174398

[B65] Steele C. M. Aronson J. (1995). Stereotype threat and the intellectual test performance of African Americans. *J. Personal. Soc. Psychol.* 69 797–811. 10.1037/0022-3514.69.5.797 7473032

[B66] Tamborini R. (2011). Moral intuition and media entertainment. *J. Media Psychol.* 23 39–45. 10.1027/1864-1105/a000031

[B67] Tamborini R. (2012). “A model of intuitive morality and exemplars,” in *Electronic Media Research Series. Media and the Moral Mind*, ed. Tamborini R., (Abingdon: Routledge), 43–74.

[B68] Thompson C. G. Kim R. S. Aloe A. M. Becker B. J. (2017). Extracting the variance inflation factor and other multicollinearity diagnostics from typical regression results. *Basic Appl. Soc. Psychol.* 39 81–90. 10.1080/01973533.2016.1277529

[B69] Thomson J. J. (1985). The trolley problem. *Yale Law J.* 94 1395–1415.

[B70] Weaver A. J. Lewis N. (2012). Mirrored morality: an exploration of moral choice in video games. *CyberPsychol. Behav. Soc. Network.* 15 610–614. 10.1089/cyber.2012.0235 23017118

[B71] WePC, (2020). *2020 Video Game Industry Statistics, Trends & Data.* Available online at: https://www.wepc.com/news/video-game-statistics/ (accessed March 16, 2020).

